# An immunoregulatory amphipathic peptide derived from *Fasciola hepatica* helminth defense molecule (FhHDM‐1.C2) exhibits potent biotherapeutic activity in a murine model of multiple sclerosis

**DOI:** 10.1096/fj.202400793RR

**Published:** 2025-02-14

**Authors:** Richard Lalor, Akane Tanaka, Jenna Shiels, Aakanksha Dixit, Sabine Hoadley, Eloïse Dufourd, Siobhan Hamon, Joyce To, Clifford C. Taggart, Sinead Weldon, Bronwyn O'Brien, Judith Greer, John P. Dalton, Sheila Donnelly

**Affiliations:** ^1^ Molecular Parasitology Laboratory, Centre of one Health (COH) and Ryan Institute, School of Natural Science University of Galway Galway Ireland; ^2^ School of Life Sciences University of Technology Sydney Sydney Australia; ^3^ Wellcome‐Wolfson Institute for Experimental Medicine Queen's University Belfast Belfast UK; ^4^ UQ Centre for Clinical Research The University of Queensland Brisbane Queensland Australia

**Keywords:** experimental autoimmune encephalomyelitis, *Fasciola hepatica*, helminth defense molecule, immune regulation, macrophage

## Abstract

The helminth defense molecules (HDM) are a family of immune regulatory peptides exclusively expressed by trematode worms. We have previously demonstrated that in vivo FhHDM‐1, the archetypal member of the HDMs, regulated macrophage responses to inflammatory ligands, thereby ameliorating the progression of immune‐mediated tissue damage in several murine models of inflammatory disease. Accordingly, we postulated that an understanding of the structure–function relationship of the HDMs would facilitate the identification of the minimal bioactive peptide, which would represent a more synthesizable, cost‐effective, potent biotherapeutic. Thus, using a combination of bioinformatics, structural analyses, and cellular assays we discovered a 40 amino acid peptide derivative termed FhHDM‐1.C2. This peptide contains a 12 amino acid motif at its N‐terminus, which facilitates cellular interaction and uptake, and an amphipathic α‐helix within the C‐terminus, which is necessary for lysosomal vATPase inhibitory activity, with both regions linked by a short unstructured segment. The FhHDM‐1.C2 peptide exhibits enhanced regulation of macrophage function, compared with the full‐length FhHDM‐1, and potent prevention of the progression of relapsing–remitting‐experimental autoimmune encephalomyelitis (EAE) when administered prophylactically or therapeutically. The protective effect of FhHDM‐1.C2 is not associated with global immune suppression, which places the HDMs peptides as an improved class of biotherapeutics for the treatment of inflammatory diseases. Comparing the HDMs from several zoonotic trematodes revealed a similar capacity for immune regulation. These important new advances into the structure–function relationship of the lead HDM peptide, FhHDM‐1, encourage further prospecting and screening of the broader trematode family of peptides for the discovery of novel and potent immune‐biotherapeutics.

## Graphical abstract

Using a combination of bioinformatics, sequence and structural analyses, we designed derivatives of the parasite‐derived helminth defense molecule to determine a relationship between the peptide structure and its immune regulatory function. Screening these derivatives in a series of assays related to the activation of inflammatory macrophages, we discovered a 40 amino acid peptide derivative termed FhHDM‐1.C2. This peptide exhibits enhanced regulation of macrophage function compared with the full‐length FhHDM‐1, and potent prevention of the progression of relapsing–remitting‐experimental autoimmune encephalomyelitis (EAE) when administered prophylactically or therapeutically. The protective effect of FhHDM‐1.C2 is not associated with global immune suppression, which places the HDMs peptides as an improved class of biotherapeutics for the treatment of inflammatory diseases.

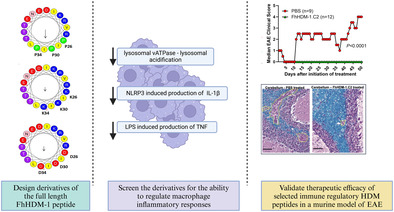

## 1 INTRODUCTION

The archetypal helminth defense molecule, FhHDM‐1, was discovered in the secretions of juvenile and adult *Fasciola hepatica* as a small molecular weight protein.^1^ While the amino acid sequence of FhHDM‐1 showed no similarity to any previously identified functional protein in public databases, bioinformatic tools predicted the presence of a helical C‐terminus containing a short amphipathic motif, which resembled that of the human defense peptide (HDP) LL‐37, and thus the HDM nomenclature was first coined.^2^ LL‐37 is a 37 amino acid peptide hydrolytically cleaved from the 18 kDa cathelicidin protein, CAP18, which is released by activated neutrophils.^3^ The LL‐37‐like HDPs are highly conserved components of the invertebrate and vertebrate innate immune system and regulate both innate and adaptive immune cell function to promote immune homeostasis.^4^, ^5^ Structure–function analysis of human LL‐37 has shown that both the α‐helical content and the charged cationic properties of the peptide were necessary for LL‐37 to inhibit pro‐inflammatory responses in human monocytes.^6^ The similarity in structure between LL‐37 and FhHDM‐1 presented the first clues regarding the putative mimetic functionality of the *F. hepatica* HDM in immune regulation.^2^ However, unlike LL‐37, FhHDM‐1 did not exhibit a strong cationic charge, which accounted for different biological properties. For example, FhHDM‐1 did not exhibit antimicrobial killing activity, and more importantly, it was not cytotoxic to mammalian cells in vitro.^7^

Exploring the biological activity of FhHDM‐1 in vivo revealed that FhHDM‐1 preferentially interacts with macrophages, embedding into the lipid rafts of the cellular membrane. Co‐localization studies showed that the peptide was internalized by macrophages once the culturing temperature was raised to 37°C and then located to endolysosomal structures suggesting uptake by active endocytosis.^8^ Once in the lysosome, cathepsin L hydrolyzed the FhHDM‐1 to release a short C‐terminal peptide (termed FhHDM‐1p2). In vitro biochemical assays have shown that this FhHDM‐1p2 inhibited the lysosomal proton pump, vacuolar(v)‐ATPase. We suggested that this targeting of vATPase by FhHDM‐1 causes elevation of lysosomal pH and impairs the functional activity of the lysosomal proteases within macrophages. Consequently, intracellular inflammatory pathways that require lysosomal acidification, such as Toll Like Receptor (TLR)‐3 or TLR‐4 signaling, or NLRP3 inflammasome activation are prevented.^9^, ^10^, ^11^ By contrast, inflammatory pathways that do not involve the lysosome remain functional in the presence of FhHDM‐1.^10^

Given the central role for pro‐inflammatory macrophages (and TLR and NLR signaling) in the initiation and progression of several immune‐mediated diseases, we proposed that FhHDM‐1‐mediated regulation of immunity could be exploited for the treatment of human diseases that result from excessive, dysregulated pro‐inflammatory responses.^12^, ^13^ Indeed, over recent years, we, and others, have shown that a short treatment course of FhHDM‐1 prevents autoimmune disease in murine models of multiple sclerosis (MS), type 1 diabetes (T1D), and rheumatoid arthritis (RA).^9^, ^13^, ^14^

We reasoned that an understanding of the structure–function relationship of the HDMs would enable the design of new peptide derivatives that are more efficacious biotherapeutics. To date, our structural analyses have shown that FhHDM‐1 has a predominantly helical structure with two α‐helices connected by a short amino acid random coil. Helical wheel analysis revealed that a region C‐terminus to this coil, which is akin to the peptide released by lysosomal cathepsin L, FhHDM‐1p2, contains an amphipathic motif.^2^ Disruption of the α‐helical structure or the amphipathic motif abolished the ability of FhHDM‐1 to inhibit vATPase. This finding suggests that the amphipathic C‐terminal region is critical for the bioactivity of the peptide.^8^ Surprisingly, however, FhHDM‐1p2 did not exert anti‐inflammatory activity on macrophages and did not significantly disrupt lysosomal function of cells.^10^ These findings implied that the α‐helix to the N‐terminus of the random coil was necessary for FhHDM‐1 binding, uptake, and delivery to the lysosome in which the bioactive C‐terminus α‐helix peptide is released.

The aim of the current study was to use our knowledge of the FhHDM‐1 structure to identify the minimal bioactive peptide by screening peptide derivatives in a range of in vitro assays that represented the mechanisms of action of FhHDM‐1 (i.e., inhibition of vATPase activity, inflammatory cytokine production, and lysosomal acidification). To validate this selection process, the identified functional peptide derivative was then tested for efficacy in vivo using a murine model of relapsing–remitting experimental autoimmune encephalomyelitis (EAE), in which we have previously demonstrated FhHDM‐1 to be an effective prophylactic.

## 2 MATERIALS AND METHODS

### 2.1 Bioinformatic analysis

Multiple sequence alignments were generated by inputting the primary amino acid sequences of the HDM peptides in Fasta format into the T‐Coffee web server and using the M‐Coffee tool; http://tcoffee.crg.cat/apps/tcoffee/do:mcoffee. Results were exported as clustalw_aln files. Amino acid sequences were entered to the I‐TASSER server^15^ (https://zhanglab.ccmb.med.umich.edu/I‐TASSER/) to obtain ab initio predictions of the secondary structure and putative 3D models of the HDM peptides. The HeliQuest tool^16^ (http://heliquest.ipmc.cnrs.fr) was used to construct helical wheel projections with analysis parameters set to alpha helical with 18 amino acid analysis windows.

### 2.2 Peptide synthesis

Peptides were synthesized to >95% purity and validated by high‐performance liquid chromatography (HPLC) and mass spectrometry (GL Biochem, Shanghai and GenScript, USA). In addition, FhHDM‐1.C1 and FhHDM‐1.C2 were synthesized as biotinylated peptides to >91% purity, and validated by mass spectrometry and HLPC (Auspep, Australia). Peptides were received lyophilized, and stored at −80°C prior to solubilization in sterile, endotoxin‐free water ready for use.

### 2.3 Culture and treatment of macrophages

Murine RAW 264.7 macrophages (American Type Culture Collection; ATCC, Manassas, USA) were cultured in R10 media (RPMI 1640 media +10% v/v heat‐inactivated fetal bovine serum [FBS]; Gibco, ThermoFisher Scientific, UK) and used at passage numbers 3–18, as recommended by the ATCC. For experimentation, cells (1 × 10^6^ cells/mL) were incubated with peptides and/or *Escherichia coli* LPS (10 ng/mL, 011:B4 strain; Sigma Aldrich, USA) in media for 16 h.

The human acute monocytic leukemia THP‐1 cell line (ATCC, Manassas, USA) was cultured (P2‐30) in RPMI 1640 medium with L‐glutamine (2 mM) (Gibco, ThermoFisher Scientific, UK) supplemented with 10% (v/v) heat‐inactivated FBS (Gibco, ThermoFisher Scientific, UK) and 1% (v/v) penicillin/streptomycin (PAA Laboratories GmbH, Pasching, Austria). For experimentation, cells were seeded at a density of 2.5 × 10^5^ cells/well in 24‐well plates and were differentiated to macrophages by incubating with 2 mL of medium with 162 nM phorbol 12‐myristate 13‐acetate (PMA; Sigma Aldrich, UK) for 72 h, then rested in fresh media (PMA‐free) for 24 h before use. Cells were then treated with peptides (20 μg/mL) and/or *Pseudomonas aeruginosa* LPS (100 ng/mL, Serotype 10, Source strain ATCC 27316; Sigma Aldrich, UK) in media for 16 h.

Macrophages were differentiated from the bone marrow of C57BL6 mice over 6 days in RPMI medium supplemented with 10%v/v FBS, penicillin/streptomycin (100 U/mL), L‐glutamine (2 mM), 2‐mercaptoethanol (2‐ME; 50 μM), and macrophage colony‐stimulating factor (M‐CSF;50 ng/mL; eBiosciences). Differentiated cells were confirmed as F4/80^+^ CD11b^+^ by flow cytometry. For experimentation, cells were seeded at a density of 1 × 10^6^ cells/well and left to adhere overnight (O/N). Cells were then washed and cultured in fresh RPMI medium with 10% FBS, penicillin/streptomycin (100 U/mL), and L‐glutamine (2 mM) with peptides and/or bacterial LPS as described for the macrophage cell lines. To induce the activation and secretion of IL‐1β, BMDMs (1 × 10^6^ cells/mL) were pre‐activated with LPS (100 ng/mL; *E. coli* 0111:B4 strain; Invivogen, FR) for 2 h, washed and cultured in fresh medium prior to 1 h treatment with peptides (4 μM) and subsequent stimulation with monosodium urate (MSU) crystals (MSU; 250 μg/mL; Invivogen, FR) for 5 h.

To confirm the stimulation of cells with LPS +/−, FhHDM‐1 peptides did not induce cell death, the release of lactate dehydrogenase (LDH) was measured using a commercially available cytotoxicity assay according to the manufacturer's instructions (Promega, UK). Figure S1 shows a representative data set from this analysis.

### 2.4 Interaction of FhHDM‐1 and FhHDM‐1.C2 peptides with macrophages

RAW 264.7 macrophages (1 × 10^6^ cells/mL) were incubated with 1 mL of biotin‐labeled peptides in PBS at 4°C for 30 min, protected from light. After washing in saline, cells were pelleted by centrifugation (300 ×g; 5 min), resuspended in 100 μL Alexa Fluor 488 labeled streptavidin (diluted 1/200 in saline; Life Technologies) and then incubated at 4°C for 30 min, protected from light. Following four washings, peptide binding to the macrophages was assessed by flow cytometry.

### 2.5 Internalization of FhHDM‐1 and FhHDM‐1.C2 peptide by macrophages

RAW 264.7 macrophages, seeded at 2 × 10^5^ cells/mL, were allowed to adhere onto 10 mm Fluorodish cell culture dishes for at least 2 h (37°C/5% CO_2_) prior to incubation with R10 media only (control untreated), or with biotin‐labeled FhHDM‐1.C2 (5 μM) for 15 min (37°C/5% CO_2_). After removal of the supernatant, cells were washed twice with PBS and then fixed with paraformaldehyde (4% v/v) at RT for 30 min. After washing with PBS three times, the cellular membranes were permeabilized by the addition of microscopy permeabilization solution (PBS + 0.1% v/v Triton‐X100; Sigma‐Aldrich, MPS) for 1 min. Cells were then washed with PBS three times prior to quenching using 100 mM glycine. Cells were again washed and then blocked with PBS supplemented with 2%v/v FBS and 0.1% v/v Tween 20 (PBS/T) O/N at 4°C. After washing, the cells were incubated with Alexa Fluor 488 labeled streptavidin (Life Technologies) diluted 1 in 20 with PBS containing 2% v/v FBS for 1 h at RT, protected from light. A cell sample incubated with Alexa Fluor 488 labeled streptavidin only was included as a control for non‐specific binding. After washing cells with PBS/T, Alexa Fluor 647 Phalloidin was used to stain the cellular membrane, or transmission detection was used to visualize the membrane. DAPI was used for nuclei staining.

For co‐localization studies, RAW 264.7 macrophages were seeded at 2 × 10^5^ cells/mL and allowed to adhere onto 10 mm Fluorodish cell culture dishes for at least 2 h (37°C/ 5% CO_2_) prior to incubation with R10 media containing 60 nM of Lysotracker (Thermo Fisher Scientific; diluted from a stock solution (in DMSO) of 1 mM) for 1 h. After three washes with PBS, Alexa Fluor 488 labeled FhHDM‐1.C2 in R10 media was added to the cells for 30 min. The culture media was removed, cells were washed three times in PBS, fixed, permeabilized, and quenched. Nuclei were stained with DAPI, as described above.

### 2.6 Determination of macrophage lysosomal pH

Changes in murine macrophage lysosomal pH induced by FhHDM‐1 and FhHDM‐1.C2 were determined using Lysosensor yellow/blue (ThermoFisher Scientific), a ratiometric lysosomal pH indicator dye. RAW 264.7 macrophages (1 × 10^6^ cells/mL) were incubated with FhHDM‐1 or FhHDM‐1.C2 (5 μM) for 30 min at (37°C/ 5% CO_2_) and then washed twice with saline prior to incubation with Lysosensor dye (5 μM) for 5 min (37°C/ 5% CO_2_). The cells were then washed with a solution of 1 × PBS with 1% w/v BSA and 2% v/v heat‐inactivated FBS containing 0.1% sodium azide to remove any excess Lysosensor dye. Cells were resuspended in 500 μL of the same solution, and then dispersed by passage through mesh into FACs tubes for flow cytometric analysis. An increase in lysosomal pH was detected as an increase in blue fluorescence, using the 450/50 nm filter.

The lysosomal pH was more accurately measured using the Lysosensor dye by comparison to a standard curve as previously described.^17^ RAW 264.7 macrophages (3 × 10^5^ cells/mL) were either untreated or incubated with FhHDM‐1, FhHDM‐1.C2, or Bafilomycin A1 (100nM, positive control) for 30 min (37°C/ 5% CO_2_). After washing with saline, cells were incubated with Lysosensor (1 μM) for 5 min, washed with PBS, and then covered in live cell imaging solution (ThermoFisher Scientific) and analyzed using a microplate reader (excitation; emission 329/440 nm and 384/540). The lysosomal pH was determined by calculating the fluorescence intensity by creating ratios of 440 nm:540 nm values and calibration using a standard curve, which was created using a separate set of macrophages that were incubated in a series of buffers (pH 3.5–7.5) for 10 mins prior to the addition of Lysosensor.

### 2.7 Isolation of macrophage lysosomal membranes and an assessment of ATPase activity

The method to prepare a lysosomal enriched fraction was adapted from Robinson et al.^8^ Briefly, RAW264.7 macrophages (2 × 10^7^) were sonicated on ice, and a post‐nuclear supernatant was centrifuged at 50 000 *g* for 20 min to pellet lysosomes and endosomes. The pellet was extracted with dH_2_O for 5 min and subsequently centrifuged at 50 000 *g* for 20 min. The resulting pellet representing the lysosome insoluble membrane fraction was resuspended in 10 mM Tris acetate (pH 7.0) overnight at 4°C and analyzed by Western blot for the lysosomal specific antibody LAMP‐1 (Abcam, Cambridge, MA, USA) to confirm the presence of lysosomes.

ATPase activity was detected in the lysosomal membrane fraction using the ATPase Assay kit (Abcam, Cambridge, MA, USA), according to the manufacturer's instructions. For inhibition assays, indicated dilutions of HDMs were pre‐incubated with the lysosome fraction in 0.1 M Tris‐buffer (pH 7.5) for 1 h at 37°C prior to incubation with the ATP substrate.

### 2.8 Cytokine analysis

Supernatants from stimulated murine macrophages were assayed for TNF, IL‐6, or IL‐1β production using cytokine‐specific ELISAs (BD Biosciences, USA, or Invitrogen, ThermoFisher Scientific, UK, respectively) according to the manufacturers' instructions. The secretion of cytokines from THP‐1 cells was quantified using human IL‐6 uncoated ELISA kit (Invitrogen, ThermoFisher Scientific, UK) or human TNF ELISA kit (Peprotech, UK), according to the manufacturers' instructions.

Mouse cytokine ELISA kits (elisakit.com, Melbourne, Australia) were used to assess levels of IFN‐γ, IL‐6, IL‐10, and TNF in supernatants of cultured lymph node cells from EAE mice treated with either PBS or FhHDM‐1.C2. Kits were used as per the manufacturer's instructions, with concentrations determined from standard curves prepared from each kit. Data are shown as fold changes in amounts of cytokine between antigen‐stimulated and unstimulated cultures.

### 2.9 EAE induction in mice and clinical and histological assessment

To induce EAE, 50 μg of myelin proteolipid protein (PLP) peptides PLP_139–151_ or PLP_178–191_, in an emulsion consisting of equal volumes of PBS and CFA containing an additional 4 mg/mL of *Mycobacterium tuberculosis* H37Ra (Difco) was injected subcutaneously (sc) into 6—8‐week‐old SJL/J mice (from in‐house breeding colony). Each mouse received 300 ng of *Bordetella pertussis* toxin (Sapphire Biosciences) intravenously (iv) on days 0 and 3. Clinical assessment (weight and observation) was conducted daily from day 7 post‐injection. Clinical signs in mice were scored according to the following criteria: 0, no disease; 1, decreased tail tone or slightly clumsy gait; 2, tail atony and/or moderately clumsy gait and/or poor righting ability; 3, hind limb weakness; 4, hind limb paralysis; and 5, moribund state. To assess the efficacy of FhHDM‐1.C2 to modulate the course of EAE, FhHDM‐1.C2 (20 μg in 100 μL sterile PBS) or PBS alone was administered by iv injection every other day for a total of six injections. Two treatment schedules were used: prophylactic administration, which commenced 2 days prior to the induction of EAE, and therapeutic administration, in which treatment was commenced at the point where mice had almost recovered (residual score of 1–2) from the first presentation of paralysis (during which time they had to have reached at least a clinical score of 3). The total observation period for mice was 70 days for mice treated prophylactically, and 50 days from the first day of treatment for mice treated therapeutically.

For histological assessment of mouse CNS tissue, PBS‐perfused brains, and spinal cords from PBS‐treated and FhHDM‐1.C2‐treated mice were fixed in 10% formalin, embedded in paraffin. Every mouse in the prophylactic treatment group was studied histologically. For the therapeutic treatment group, five mice from the PBS group were randomly selected for histology, and each of these was paired with a FhHDM‐1.C2‐treated mouse that had commenced treatment on the same day. Sagittal sections (8 μm) of paraffin‐embedded brain and spinal cord tissue were cut and stained with Luxol fast blue and hematoxylin and eosin, to visualize myelination and inflammation, respectively. Two to four tissue sections (sections cut approximately 100 μM apart) from each mouse were then scanned using a 3D Histech slide scanner (Budapest, Hungary), and viewed using QuPath software.^18^ Images at the same magnification of the cerebellum and spinal cord were assessed for the percentage of the total myelinated region (i.e., LFB‐stained area) that contained inflammatory cells and/or demyelination using ImageJ software. The averages of all sections from the same region from each individual mouse were used to generate the figures shown.

### 2.10 T‐cell proliferation assays

Lymph nodes were removed from PBS‐ or FhHDM‐1‐treated mice at times outlined in the text. Lymph node cells were labeled with cell‐trace violet (CTV, ThermoFisher Scientific) and stimulated in vitro without antigen or with either myelin peptides (PLP_178–191_, PLP_215–232_, Myelin Oligodendrocyte Glycoprotein (MOG)_35–55_), FhHDM‐1.C2 or Concanamycin A (ConA) for 3 or 6 days. Cells were then harvested, labeled with PE‐labeled anti‐CD4 antibody and assessed for proliferation of CD4^+^ T cells (indicated by decreased CTV staining) by flow cytometry on a Beckman Coulter Gallios Flow Cytometer. Data were analyzed using FlowJo™. The cell division index (CDI) was determined from the percentage of cells proliferating in response to antigen divided by the percentage of proliferating cells in the without antigen control group.

### 2.11 Ethical approval

Ethical approval for the isolation of BMDMs was granted by the University of Technology Sydney (UTS) Animal Care and Ethics Committee (Approval Number: 2017–1232), and experiments were conducted in accordance with approved guidelines compliant with The Australian Code for the Care and Use of Animals for Scientific Purposes. In addition, ethical approval for the use of animal tissues in these studies was reviewed and approved by the University of Galway Animal Care Research Ethics Committee (ACREC Approval Number: 19‐Nov‐02), and experiments were conducted in accordance with ARRIVE 2.0 guidelines for best practices for the use of animals for scientific purposes. Ethical approval for experiments involving the EAE murine model was granted by the University of Queensland Animal Ethics Committee (Approval Numbers: 434‐17 and 2022/AE000128), and all protocols were conducted in accordance with the approved guidelines.

### 2.12 Statistical analysis

Data analysis was conducted using Microsoft Excel (Microsoft Windows) and GraphPad Prism (GraphPad, CA, USA) with statistical analysis conducted using ANOVA or student *t*‐test as detailed in the figure legends. Differences were not deemed significant when *p*‐values (*p*) > .05. For comparing the clinical course data in the EAE study, two‐way repeated measures ANOVA tests were used when there were no missing values, and a mixed‐effects model with the Geisser–Greenhouse correction was used if there was missing data (e.g., if a mouse died during the follow‐up period).

## 3 RESULTS

### 3.1 The sequence around the C‐terminal helix of FhHDM‐1 contains its biological activity

**FIGURE 1:**
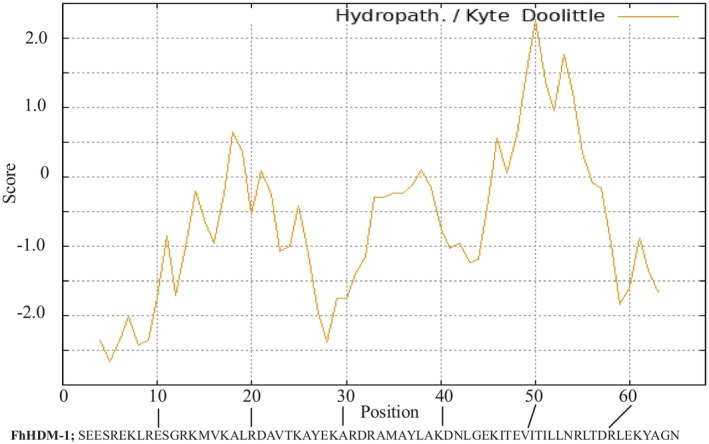
Hydrophobicity plot and cell‐penetrating peptide probability analysis of FhHDM‐1. Kyte & Doolittle plots were used to determine the hydrophobicity of regions of an amino acid sequence based on calculations using a score of hydrophobicity for the constituent amino acids. A higher score (y‐axis) denotes a more hydrophobic that region of the molecule (x‐axis).

To first explore the possibility that specific regions of the FhHDM‐1 peptide could mediate cellular uptake, the hydropathy of the amino acid sequence was determined using a Kyte & Doolittle plot (Figure 1), as the presence of a relatively hydrophobic moiety, regardless of structure, is a key determinant in the capacity of a peptide to cross cellular membranes.^19^, ^20^ This analysis revealed that the FhHDM‐1 peptide was largely hydrophobic, particularly toward the C‐terminal end. However, the hydrophobicity plot also identified a notable change in the properties of FhHDM‐1 around position 29, which was considerably hydrophilic. This profile indicated that the C‐terminus region spanning from the lysine residue at position 29 has a hydropathy profile associated with an enhanced capacity to penetrate cell membranes.

Using this information, a series of FhHDM‐1 peptide derivatives were synthesized to determine the minimal FhHDM‐1 peptide sequence that mediated the immune regulatory effect on macrophages (Table 1). The design of these derivatives was based on our predicted structure of the HDM peptides, and the position of hydrophilic and hydrophobic regions, and comprised the N‐terminus (FhHDM‐1.N), the middle region (FhHDM‐1.M), and the C‐terminus amphipathic region (peptides FhHDM‐1.C1 to C5). The FhHDM‐1.C1 peptide represented the region that contains the amphipathic helix that had been previously characterized as exhibiting vATPase inhibitor activity (FhHDM‐1.C1; previously termed FhHDMp2 in Robinson et al., 2011^2^). Alternative peptides from the C‐terminus were designed to contain the vATPase inhibitor activity with either additional amino acids from the N‐terminus (FhHDM‐1.C2 to C4) around position 29, or with a truncated C‐terminal end (FhHDM‐1.C5).

**TABLE 1:**
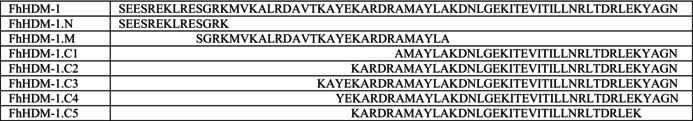
Peptide derivatives designed from the full‐length FhHDM‐1 sequence.

**FIGURE 2:**
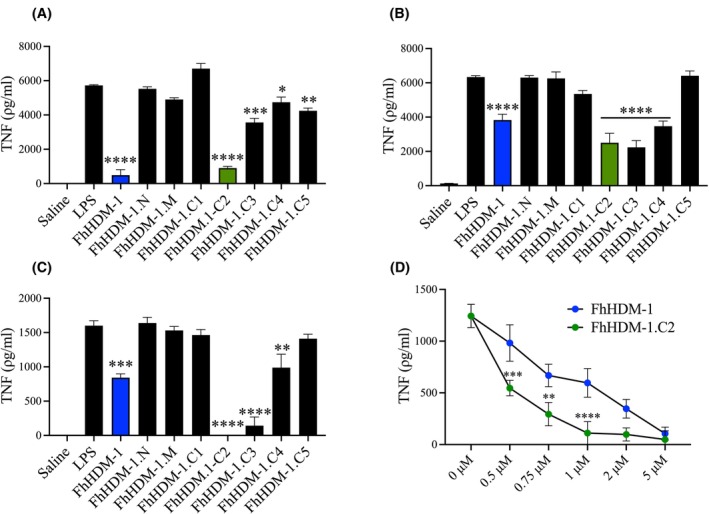
The FhHDM‐1.C2 is the shortest derivative of full‐length FhHDM‐1 to significantly suppress macrophage inflammatory responses. (A) THP‐1, (B) RAW 264.7, and (C) BMDM macrophages were treated FhHDM‐1 or the peptide derivatives for 1 h prior to stimulation with LPS for 16 h. Secretion of TNF in the supernatant was quantified by ELISA using cell supernatants. Data presented as the mean ± SEM are representative of three independent experiments. The significance of differences in the quantity of TNF secreted by HDM‐treated cells in comparison to LPS only, was determined by ANOVA with Dunnett's multiple comparison test; (D) Dose response curves were generated by treating BMDM macrophages with FhHDM‐1 and FhHDM‐1.C2 (0.5–5 μM) for 1 h prior to overnight stimulation with LPS. Levels of TNF in the supernatant was quantified by ELISA. Data are presented as the mean ± SD and are representative of three independent experiments analyzed using ANOVA with Tukey's multiple comparison test. *p < .05, **p < .01, ***p < .001, ****p < .0001.

Initial screening of the bioactivity of the FhHDM‐1 peptide derivatives examined their ability to inhibit TNF production by both murine and human macrophages stimulated with bacterial LPS. We chose to compare the immune regulatory activity of the FhHDM‐1 peptides in the broadly accepted cell lines of murine RAW 246.7 macrophages and human THP‐1 monocytes. Furthermore, to validate the outcomes from this screening, and thus support the transition to in vivo models of disease, we also compared the regulatory effect of the peptides in primary murine macrophages. Thus, human THP‐1, murine RAW 264.7, and murine bone marrow‐derived (BMDM) macrophages were treated with FhHDM‐1, and the various peptide derivatives, for 1 h prior to stimulation with LPS. As expected, LPS alone significantly increased TNF production, which was significantly reduced when cells were pre‐treated with the full‐length FhHDM‐1 (Figure 2A–C). Peptides lacking some (FhHDM‐1.C5) or all (peptides N and M) of the amphipathic helix showed no significant inhibitory effect on the production of TNF by macrophages in response to inflammatory stimuli. Also, in agreement with previous cellular assays,^8^, ^10^ the amphipathic FhHDM‐1.C1 did not effectively suppress the induction of TNF in LPS stimulated macrophages.

By contrast, all peptides containing both the full amphipathic helix and additional amino acids from the N‐terminus (FhHDM‐1.C2‐C4) significantly inhibited TNF production (Figure 2A–C). Comparisons of concentrations of FhHDM‐1 and the shortest active peptide, FhHDM‐1.C2 (Figure 2D) revealed significant differences in suppressive activity at lower concentrations (*p* = .0003, .017 and .0001 for 0.5, 0.75, and 1 μM, respectively). Collectively, these data show that FhHDM‐1.C2 exerted a superior inhibitory effect, as compared with the full‐length FhHDM‐1 peptide.

### 3.2 FhHDM‐1.C2 binds to, and is internalized by macrophages and localizes to the lysosome

While our previous observation that the FhHDM‐1.C1 derivative inhibited lysosomal vATPase indicated that this was a functional peptide, the comparison of regulatory activity in macrophages between FhHDM‐1.C1 and the longer FhHDM‐1.C2 supports our hypothesis that an amino acid sequence to the N‐terminus of the FhHDM‐1.C1 region is required to translate the peptide bioactivity to a cellular system. Corroborating this view was the prediction by Martínez‐Sernández et al.,^21^ that the N‐terminal sequence of FhHDM‐1 (position 5–33) was a cell‐penetrating peptide (CPP). Cell‐penetrating peptides are short peptides containing protein transduction domains that facilitate the transport of molecular cargo across cellular membranes, resulting in the internalization of their cargo with limited toxicity.^22^, ^23^ Accordingly, the presence of such a motif would explain why the N‐terminal‐extended FhHDM‐1.C2 peptide exhibited superior immune regulatory activity in macrophages, as compared with the shorter FhHDM‐1.C1. In addition, the observation that FhHDM‐1.C3‐C4 were slightly less inhibitory than FhHDM‐1.C1 in macrophages suggests the presence of a short CPP motif rather than a requirement for the entire N‐terminus.

To investigate the possible identity of this CCP region, the sequences of FhHDM‐1 and the C‐terminal derivatives were assessed by the predictive software, MLCPP 2.0^24^ (Table S1), which ranks peptides from 0 to 1, according to predictive algorithms that determine the probability that a peptide is cell‐penetrating. Scores closer to 1 indicate confidence that the peptide will be cell‐penetrating, while scores closer to 0 suggest that the peptide is highly unlikely to be cell‐penetrating. This analysis attributed the highest predictive score (0.974) to a region of 12 amino acids (KARDRAMAYLAK) positioned to the N‐terminus of the FhHDM‐1.C1 comprising an additional 5 amino acids (KARDR) to the FhHDM‐1.C1 sequence. Accordingly, the data suggest that this short sequence (position 29–40) represents a CPP motif and was likely mediating the enhanced uptake, and thus bioactivity of the longer C2 peptide derivative of FhHDM‐1. Extending this sequence beyond the 12 amino acid motif toward the N‐terminus (i.e., FhHDM‐1.C3‐C4) reduced the CCP score. Predictive modeling of the 3D structure of FhHDM‐1 by AlphaFold (https://alphafold.ebi.ac.uk/entry/F6KNY7) suggests that this CPP motif is located at the end of the N‐terminal α‐helix adjacent to the short coil that forms the structural break between the C and N‐terminal α‐helices, revealing a relationship between the structure and functional activity.

**FIGURE 3:**
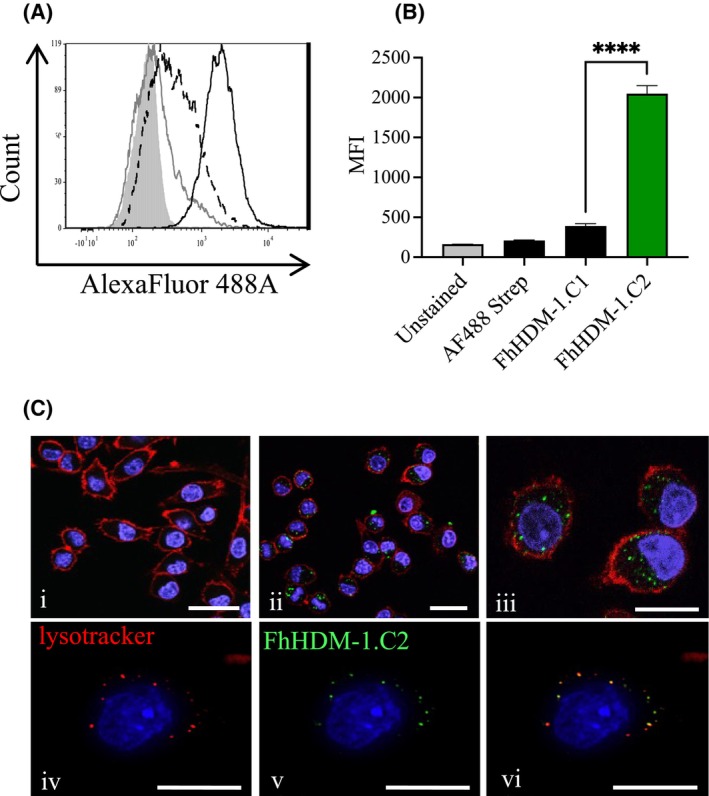
The FhHDM‐1.C2 peptide derivative binds to macrophages, is internalized and localizes to the lysosome. (A) RAW 264.7 macrophages were either unstained (gray filled) or incubated with biotin‐labeled FhHDM‐1.C1 (dotted line) or FhHDM‐1.C2 (Black line) peptides at 4°C for 30 min. To quantify peptide binding, an AlexaFluor 488‐conjugated streptavidin was subsequently added to the cells (gray line) and (B) changes in MFI were recorded by flow cytometry. Data presented are the mean in MFI ± SEM, and is representative of three independent experiments, analyzed using ANOVA with Tukey's multiple comparison test; ****p < .0001. (C; i–iii) RAW 264.7 macrophages were either (i) untreated, or (ii and iii) incubated with biotin‐labeled FhHDM‐1.C2 at 37°C for 15 min. Addition of AlexaFluor 488‐conjugated streptavidin allowed visualization (green) of the intracellular peptide by laser scanning confocal microscopy (LSCM). Images are representative of six fields of view, from five independent experiments; (C; iv–vi) Co‐localization (yellow) of FhHDM‐1.C2 (green) with LysoTracker (red) shows that FhHDM‐1.C2 enters the endolysosmal pathway in macrophages. Images are representative of seven fields of view. Scale bars = 12 μm.

To verify that the addition of this motif was enhancing cellular interaction and uptake of the HDM peptide derivatives, we compared the capacity of FhHDM‐1.C1 and FhHDM‐1.C2 to bind to cellular membranes in vitro. Addition of labeled FhHDM‐1.C1 to macrophages resulted in a small increase in their fluorescence, as compared with controls. Conversely, the addition of FhHDM‐1.C2 resulted in a much greater increase in total cell fluorescence (Figure 3A). The difference between the peptides' binding capabilities was demonstrated by the MFIs of macrophages incubated with FhHDM‐1.C1, as compared with FhHDM‐1.C2 treated macrophages (390 ± 30 and 2048 ± 103, respectively, *p* < .0001) (Figure 3B). These data show that FhHDM‐1.C2 possesses an enhanced capacity to interact with macrophages, in comparison to the shorter, CPP‐motif deficient, FhHDM‐1.C1 peptide.

To determine if the 40 amino acid derivative FhHDM‐1.C2 could penetrate the macrophage membrane, RAW 264.7 macrophages were incubated at 37°C with biotin‐labeled FhHDM‐1.C2, after which the addition of Alexa Fluor 488‐conjugated streptavidin was used to localize the peptide. Internalization of FhHDM‐1.C2 occurred after 15 min, as determined by the presence of green punctate fluorescence within the cytoplasmic region of macrophages (Figure 3Cii,iii), which was absent in the untreated controls (Figure 3Ci). Quantitative analysis determined that 99.6 ± 0.4% of cells had internalized FhHDM‐1.C2 and co‐localization of FhHDM‐1.C2 with lysotracker (red fluorescence) proved that the peptide entered the macrophage endolysosomal/lysosomal pathway (Figure 3Civ–vi).

### 3.3 Following internalization FhHDM‐1.C2 increases the lysosomal pH of macrophages to a greater extent than the full‐length FhHDM‐1

**FIGURE 4:**
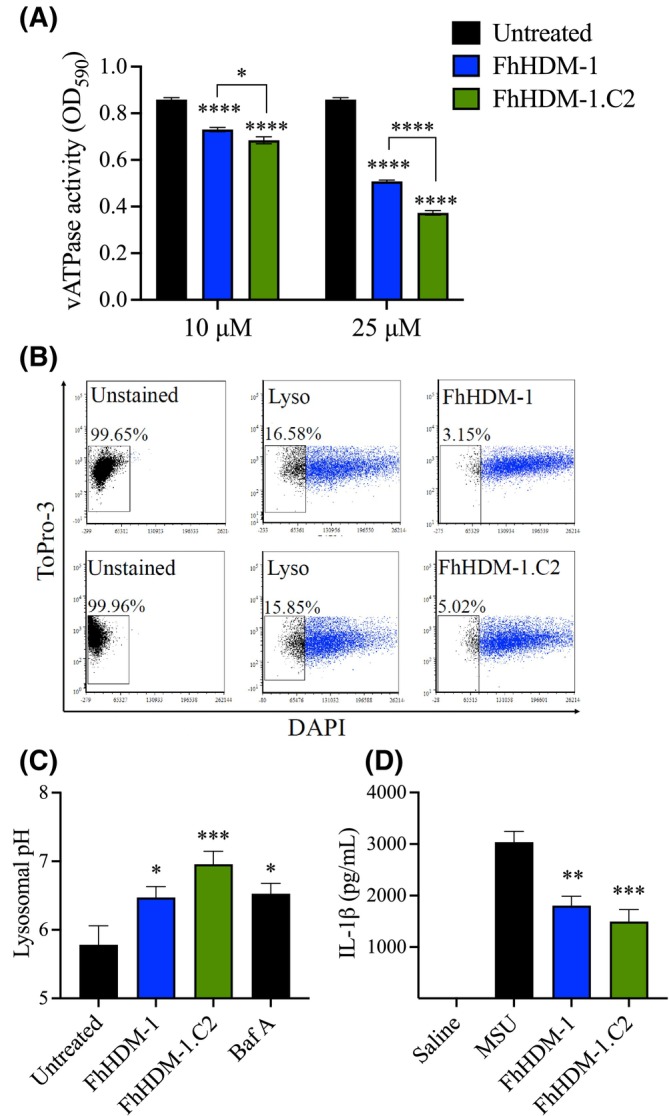
The FhHDM‐1.C2 is more effective than FhHDM‐1 in altering the lysosomal pH via vATPase inhibition. (A) ATPase activity associated with lysosomal membranes prepared from RAW 264.7 macrophages and then either untreated or treated with FhHDM‐1 or FhHDM‐1.C2 was quantified using an ATPase assay kit. Data are presented as the mean ± SD of four independent experiments analyzed using two‐way ANOVA comparison test. (B) RAW 264.7 macrophages were incubated with FhHDM‐1 or FhHDM‐1.C2 for 30 min at 37°C. Lysosensor yellow/blue was then added to each sample for 5 min and the emission of blue fluorescence (DAPI) measured at 450/50 nm (x‐axis). To allow the quantification of increased blue fluorescence, a gate was created based on the distribution of the unstained population. A decrease in the percentage of cells in this gate reflected an increase in blue fluorescence (and thus an increase in lysosomal pH). The images are representative of 5–13 independent replicates. (C) The lysosomal pH of macrophages was determined by calculating the fluorescence intensity by creating ratios of 440 nm:540 nm after the addition of Lysosensor yellow/blue and calibration using a standard curve. Data are presented as the mean ± SEM of four independent experiments and significant differences of HDM treatments compared with untreated macrophages determined using ANOVA with Dunnett's multiple comparison test. (D) The activation of NLRP3 inflammasome was determined by priming BMDMs with LPS (100 nM) for 2 h prior to incubation with saline, FhHDM‐1 or FhHDM‐1.C2 (5 μM) for 1 h and subsequent stimulation with monosodium urate (MSU) crystals (250 μg/mL) for 5 h. The level of IL‐1β in the supernatant was quantified by ELISA. Data are presented as the mean ± SD of three independent experiments and significant differences of HDM treatments compared with MSU treatment only, determined using ANOVA with Dunnett's multiple comparison test. *p < .05, **p < .01, ***p < .001, ****p < .0001.

Biochemical analysis has previously shown that the full‐length FhHDM‐1 and FhHDM‐1.C1 inhibited the vATPase activity of lysosomal membrane fractions isolated from macrophages. However, this effect did not translate to cellular assays, in which the activation of lysosomal proteases was not effectively inhibited by FhHDM‐1.C1.^8^ The enhanced binding and uptake of the FhHDM‐1.C2 peptide, in comparison to the FhHDM‐1.C1, suggested that this longer C‐terminal derived peptide facilitated the intracellular activity of the full‐length FhHDM‐1. Therefore, the capacity of HDMs to alter vATPase activity, lysosomal pH, and the production of IL‐1β was compared. As expected, both the full‐length FhHDM‐1 and the FhHDM‐1.C2 derivative peptide significantly inhibited the activity of lysosomal ATPase (Figure 4A). However, reflecting the comparative inhibition of TNF production, at both concentrations tested (10 μM and 25 μM), the FhHDM‐1.C2 was significantly more potent than the parent FhHDM‐1 peptide. This enhanced activity was also evident in measures of lysosomal pH in live macrophages as determined by flow cytometry. RAW 264.7 macrophages were incubated with FhHDM‐1 or FhHDM‐1.C2, washed twice with saline to remove excess peptide, and then incubated with Lysosensor, a ratiometric probe that produces blue fluorescence in neutral and alkaline environments and yellow fluorescence in acidic environments. To exclude interference from dead cells, TOPRO‐3 was added to the wells immediately prior to data acquisition. A gate was created for the unstained population to ensure that decreases in the percentage of cells within this gate reflected an increase in blue fluorescence (and thus an increase in lysosomal pH). Approximately 17% of cells not treated with HDM peptides, but stained with Lysosensor, populated this gate (Figure 4B), representing the baseline fluorescence of dye alone. By contrast, within this gate, there was a decrease in the percentage of cells in samples that were incubated in the presence of FhHDM‐1 or FhHDM‐1.C2 (Figure 4B; ~ 3% and 5% for FhHDM‐1 and FhHDM‐1.C2, respectively) representing an increase in blue fluorescence, and therefore, an alkaline lysosomal environment.

Construction of a calibration curve using a range of pH buffers enabled a specific measurement of lysosomal pH. For comparison, and to validate the technique, cells were also treated with Bafilomycin A, a well‐characterized vATPase inhibitor (Figure 4C). As anticipated, the pH of macrophage lysosomes was increased from an average of 5.78 (± 0.3) in untreated cells to an average of 6.5 (± 0.2) following exposure to Bafilomycin A. A similar pH increase was observed in cells treated with the full‐length FhHDM‐1, with an average pH of 6.3 (± 0.2). Nonetheless, treatment of macrophages with the FhHDM‐1.C2 raised the pH of lysosomes to an average of 7.0 (± 0.2), revealing a significantly enhanced potency of this derivative, as compared with the full‐length FhHDM‐1 peptide (*p* = .02).

These changes in vATPase activity and lysosomal pH induced by the HDM peptides correlated with the reduced secretion of IL‐1β by macrophages in response to MSU crystals as compared with untreated cells (Figure 4D). Exposure of cells to a combination of bacterial LPS and MSU stimulates the oligomerization of the NLRP3 inflammasome, via the activation of lysosomal cysteine proteases, which requires an acidic pH.^25^ Activation of NLRP3 initiates the processing and release of IL‐1β. The alkalization of lysosomes inactivates the resident cysteine proteases, thereby preventing the NLRP3 activation and IL‐1β release.^23^ Thus, the significant inhibition of vATPase (Figure 4A) and a resultant increase in pH (Figure 4B,C) explains the means by which FhHDM‐1 and FhHDM‐1.C2 significantly suppressed the production of IL‐1β by the NLRP3 inflammasome (Figure 4D).

**FIGURE 5:**
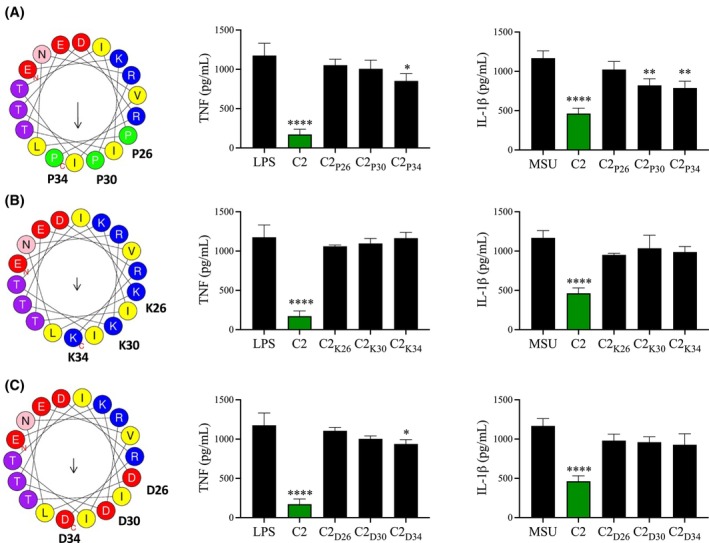
Disruption of the amphipathic region of the FhHDM‐1.C2 peptide removes the capacity to regulate macrophage inflammatory responses. Analysis of the HDMs using the Heliquest bioinformatic tool identified an amphipathic helix in the C‐terminal region of FhHDM‐1. Replacing leucine at positions 26, 30 and 34 with (A) proline, (B) lysine or (C) aspartic acid prevented FhHDM‐1 induced suppression of the macrophage pro‐inflammatory responses. Effects of LPS stimulation of macrophages were determined by the quantification of TNF production from BMDMs that had been treated with saline or HDM‐1.C2 derivatives (1 μM) for 1 h prior to stimulation with LPS (100 nM) for 18 h. Effects on monosodium urate (MSU) crystals induced inflammasome activation were determined by the quantification of IL‐1β production from BMDMs that had been pre‐activated with LPS (100 nM) for 2 h and subsequently treated with saline or HDM‐1.C2 derivatives (4 μM) for 1 h prior to stimulation with MSU (250 μg/mL) for 5 h. The levels of cytokines in the supernatant were quantified by ELISA. Data are presented as the mean ± SD and are representative of three independent experiments analyzed using ANOVA with Dunnett's multiple comparison test. *p < .05, **p < .01, ****p < .0001.

This immune regulation of macrophages by *F. hepatica* FhHDM‐1.C2 was dependent on a functional amphipathic α‐helix as substitution of the hydrophobic leucines at positions 26, 30, and 34 in the center of the hydrophobic patch, with either proline, lysine, or aspartic acid (thus changing the structure and/or charge), abrogated the ability to regulate LPS‐induced inflammation, MSU‐induced IL‐1β production (Figure 5), and the inhibition of vATPase activity (Figure S2). These results and observations are consistent with our previous in vitro mutational analysis of full‐length FhHDM.^2^

### 3.4 FhHDM‐1.C2 prevents the progression of relapsing–remitting experimental autoimmune encephalomyelitis (EAE) when administered after onset

**FIGURE 6:**
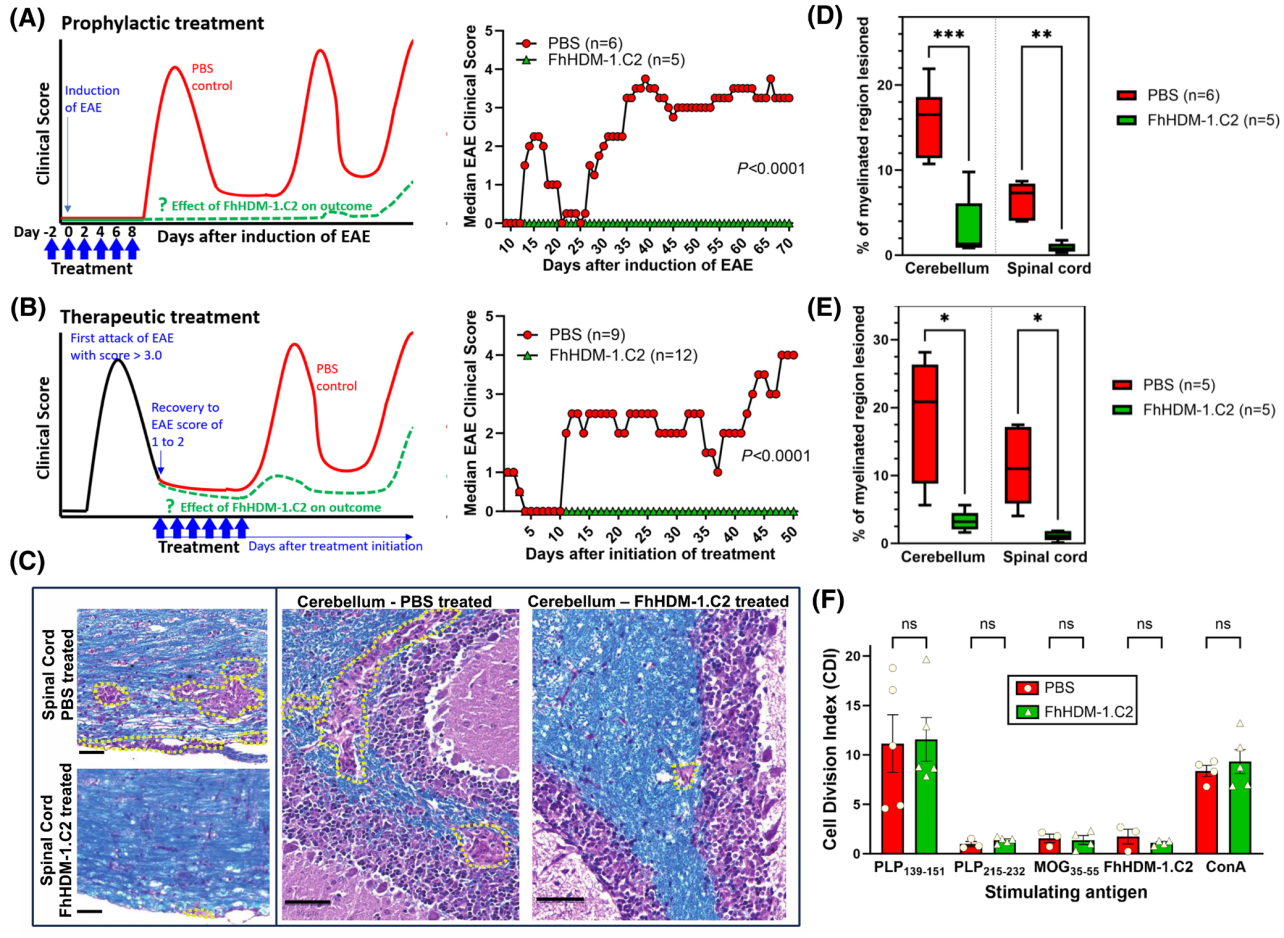
FhHDM‐1.C2 displays potent therapeutic efficacy in a murine model of relapsing–remitting multiple sclerosis but does not inhibit encephalitogenic T‐cell reactivity. (A) Schematic showing the treatment schedule (left) and results (right) for mice treated prophylactically with PBS or FhHDM‐1.C2. Because the mice do not all relapse synchronously, the median EAE score of all animals at each day is shown. (B) Schematic showing the treatment schedule (left) and results (right) for mice treated therapeutically with PBS or FhHDM‐1.C2. The median EAE score of all animals at each day is shown. (C) Representative sections of Luxol fast blue/hematoxylin and eosin‐stained cerebellar (top) or spinal cord (bottom) tissue from mice treated with PBS or FhHDM‐1.C2. Luxol fast blue stains myelin blue. Inflammatory lesions are outlined with dotted yellow lines. (D and E) Enumeration of the total percentage of the spinal cord or cerebellar white matter (myelinated regions) that was covered by lesions. Each circle or triangle represents the average percentage lesioned of 2–4 sections from an individual mouse. (D) Every mouse in the prophylactic treatment group was studied histologically. (E) For the therapeutic treatment group, five mice from the PBS group were randomly selected for histology, and each of these was paired with a FhHDM‐1.C2‐treated mouse that had treatment commenced on the same day as the PBS‐treated mouse. (F) Comparison of CD4^+^ T cells from the PBS‐ and FhHDM‐1.C2‐treated mice revealed no differences in their ability to response to the encephalitogenic peptide used to induce EAE in the mice (PLP_139–151_), or other control peptides (PLP_215–232_ and MOG_35–55_), or the FhHDM‐1.C2 peptide, or the T‐cell mitogen Concanavalin A (Con A), indicating that the protective effects of the FhHDM‐1.C2 peptide do not involve inhibition of encephalitogenic T cells. *p < .05, **p < .01, ***p < .001.

We have previously demonstrated that the immune regulatory properties of FhHDM‐1, translated to an in vivo model of immune‐mediated disease and protected against the development of EAE in mice.^9^ However, in those studies, FhHDM‐1 was delivered to mice during the induction process of EAE, and therefore, investigated as a prophylactic rather than as a treatment for disease. In the present study, we examined whether the derivative peptide FhHDM‐1.C2 could mimic the effect of FhHDM‐1 prophylactically, and operate as an effective immune regulatory biotherapeutic. Thus, we utilized the relapsing–remitting model of EAE (SJL/J mice immunized with myelin proteolipid protein peptide 139–151; PLP_139–151_) which in contrast to the more commonly used model of acute monophasic paralysis, more closely replicates the clinical phases of human multiple sclerosis (MS).^26^ For most people, MS will initially follow a relapsing–remitting pattern (RRMS) in which the formation of new lesions and the development of new clinical signs or symptoms is followed by a period of remission in which there is full or partial recovery with resolution of inflammation and evidence of re‐myelination. The murine RRMS model therefore offers opportunities to better mimic the delivery of treatment options in people living with MS. FhHDM‐1.C2 or PBS (as a control) was administered just prior to induction of EAE (prophylactic regime) or after mice had recovered from their first episode of paralysis, during which they had reached a clinical score of at least 3 (tail atony and bilateral hind limb weakness) (Figure 6A,B). The same administration regime as used in the previous assessment of FhHDM‐1 efficacy was followed^9^ in which peptide or saline were delivered by intravenous injection to groups of mice on alternate days over a total of six treatments (20 μg each), and mice were subsequently monitored for ~ 50 days (i.e., 70 days after the induction of EAE). We found, as expected, that 100% of PBS‐treated control mice developed EAE irrespective of the timing of the PBS delivery (Figure 6A,B). In contrast, mice treated with FhHDM‐1.C2 either via prophylactic or therapeutic protocols exhibited significantly (*p* < .0001) less severe disease (Figure 6A,B).

In the prophylactic treatment group (Figure 6A), only one of the FhHDM‐1.C2 treated mice developed EAE during the 10 weeks in which they were followed, reaching a maximum clinical score of 2 on day 65 after the induction of EAE; none of the other FhHDM‐1.C2 treated mice showed any clinical signs of EAE, whereas the PBS‐treated group reached a mean maximal clinical score of 4.3 ± 0.3. To confirm these results using a different inducing antigen, we initiated disease with myelin proteolipid protein peptide 178–191 (PLP_178–191_), rather than PLP_139–151_, which was used in the previous experiments. Similar to that observed previously, 6/6 mice treated with PBS reached a maximal clinical score of 3.0 ± 0.4, whereas only 4/7 mice treated with FhHDM‐1.C2 developed very mild EAE (maximal clinical score of 1.0 ± 0.4; *p* < .05 vs. PBS‐treated group). By showing that FhHDM‐1.C2 was equally potent therapeutically in EAE models induced by either PLP peptide, we have demonstrated that the protective effect is not restricted to a single PLP derivative.

For the therapeutic treatment group, EAE was induced with PLP_139–151_ in 24 mice and those that reached a clinical score of >3.0 (*n* = 21) were randomized to receive PBS or FhHDM‐1.C2 once they had started to recover from the first attack of disease and their clinical score had reduced to between 1 and 2. Mice receiving PBS subsequently developed, on average 2.8 ± 0.4 relapses, with a mean maximal clinical score of 3.7 ± 0.2 during those relapses. Mice given FhHDM‐1.C2 exhibited significantly fewer relapses of EAE (0.7 ± 0.2; *p* < .0001 vs. PBS‐treated group) and a significantly lower maximal clinical score during the relapses (1.2 ± 0.4; *p* = .0002 vs. PBS‐treated group) (Figure 6B).

Histological analysis of brain and spinal cords at the end of the experimental period (day 50 after commencement of therapeutic treatment) showed that as expected, EAE mice treated with PBS had extensive damage to white matter tracts throughout the CNS. By contrast, the beneficial impact of the peptide (delivered either prophylactically or therapeutically) was clearly evident in the reduced pathology observed in the cerebellum and spinal cord of the FhHDM‐1.C2‐treated mice (Figure 6C–E). Also apparent was the presence of far less inflammation and a lack of demyelination in the cerebellum of peptide‐treated mice. Although small accumulations of inflammatory cells were evident in the brain tissue of the peptide‐treated mice, these were generally contained within the basement membrane around the perivascular space (Figure 6C).

In the EAE model, disease is initiated by CD4^+^ T cells that are specific for the PLP peptide used to induce the disease.^27^ Therefore, to measure the impact of FhHDM‐1.C2 treatment on this population of immune cells, we measured the proliferative ability of CD4^+^ cells from lymph nodes harvested from EAE mice (*n* = 3–5 per group) that had been culled 3 days after the final prophylactic treatment of either PBS or FhHDM‐1.C2, or at the end of the observation period (~day 70; *n* = 4/group). These CD4^+^ T cells were tested for their ability to respond to the encephalitogenic inducing peptides (PLP_139–151_ or PLP_178–191_), or other non‐specific control myelin peptides (PLP_215–232_ and MOG_35–55_), the FhHDM‐1.C2 peptide, or the T‐cell mitogen Concanavalin A (Con A). The result revealed no significant differences in the cell division index (CDI) between lymph node cells from the PBS and FhHDM‐1.C2 treatment groups in response to any of the antigens. Although the CDIs in response to the inducing antigen (PLP_139–151_) were lower at the later timepoint, as might be expected ~ 10 weeks after disease initiation, there were still no significant differences between the PBS and FhHDM‐1.C2‐treated groups. Furthermore, quantification of cytokines in the supernatant of lymph node cells stimulated in vitro with no antigen, PLP_178–191_, FhHDM‐1.C2 or ConA for 3 days, showed no significant differences in the production of IFN‐γ, IL‐6, IL‐10, or TNF in cells (Figure S3). Collectively, these results indicate that the protective effects of the FhHDM‐1.C2 peptide in EAE occur independently of any effect on the activation of encephalitogenic T cells.

To further support this finding, we treated mice (3/group) with either PBS or FhHDM‐1.C2 according to the prophylactic protocol and then induced EAE with PLP_178–191_. On day 10 after induction of EAE, just prior to the onset of clinical signs, spleens were removed from the treated mice and activated in vitro with 2 μg/mL ConA for 2 days. White cells were then isolated and administered intravenously into naïve mice (5 × 10^6^ cells per mouse, 5 mice per group) that were then followed for the development of EAE. All treated mice developed mild clinical signs of EAE (confirmed by an independent, blinded observer) with no significant differences in disease development observed in mice that received cells from PBS‐treated mice or FhHDM‐1.C2 peptide‐treated mice (Table S2).

### 3.5 The HDM.C2 peptide homologues from related trematodes exhibit conservation of structure and variability in immune regulatory activity

**FIGURE 7:**
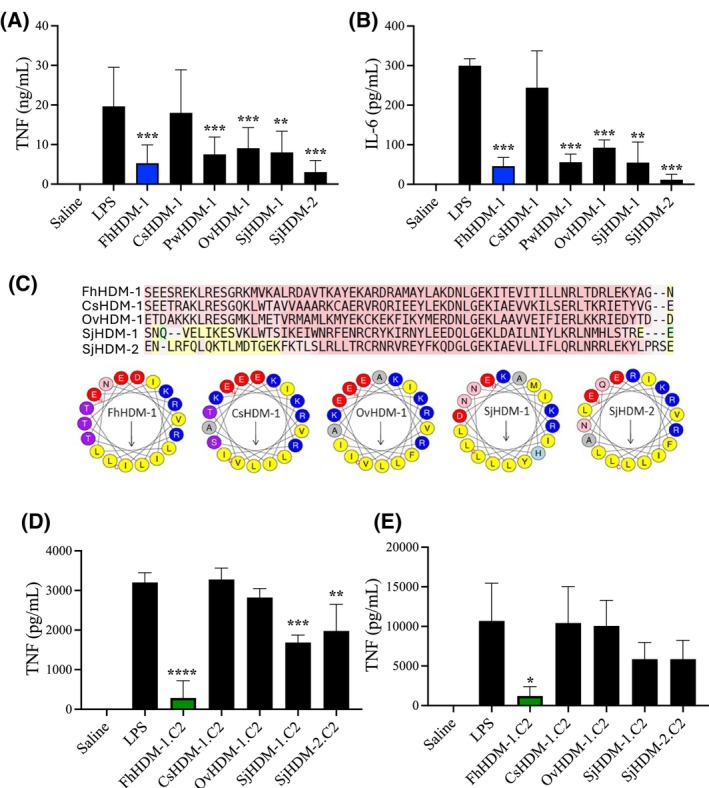
C‐terminal amphipathic HDM homologues from related trematode species do not exhibit the same anti‐inflammatory potency as FhHDM‐1.C2. Levels of (A) TNF and (B) IL‐6 were measured by ELISA, in THP‐1 macrophages stimulated with 100 ng/mL LPS for 16 h with or without FhHDM‐1, CsHDM‐1, PwHDM‐1, OvHDM‐1, SjHDM‐1, and SjHDM‐2. (C) Sequence alignments of trematode‐specific HDMs generated with T‐Coffee. Sequences highlighted in red, yellow, or green are indicative of regions of high, medium, or low structural homology respectively. The Heliquest bioinformatic tool identified an amphipathic helix in the C‐terminal region all trematode peptides, highlighted in yellow. (D) BMDMs and (E) THP‐1 cells were treated with saline or HDM‐1.C2 derivatives (1 μM) from various trematodes species for 1 hr prior to stimulation with LPS (100 nM) for 18 h. The level of TNF in the supernatant was quantified by ELISA. All data are presented as the mean ± SD and are representative of three independent experiments analyzed using ANOVA with Tukey's multiple comparison test. *p < .05, **p < .01, ***p < .001, ****p < .0001.

We have previously reported multiple sequence alignments, which showed that the full‐length HDMs expressed by different digenean trematodes (including those of the genus Schistosoma, Clonorchis and Opisthorchis) exhibit a high degree of conservation.^2^, ^28^ To determine if the immune regulatory activity demonstrated by FhHDM‐1 is also conserved, synthetic versions of a selection of HDM peptides from different parasites were tested for their ability to modulate the macrophage response to inflammatory ligands in vitro. This analysis revealed a similar capacity for the regulation of macrophage responses, with all HDMs (except the HDM derived from *Clonorchis sinensis*) significantly reducing the production of TNF and IL‐6 by human (THP‐1) macrophages in response to LPS stimulation (Figure 7A,B).

An examination of the amino acid sequence homology of these peptides shows that the C‐terminal region of the peptide is particularly conserved (Figure 7C), suggesting a common and preserved biological activity for this region. In addition, helical wheel projections (using the HeliQuest bioinformatic tool) of the primary sequences of the various trematode HDMs revealed that each displays a “patch” of hydrophobic amino acid residues that concentrate on one face of the C‐terminal α‐helix after a short, conserved coil (Figure 7C). The hydrophobic moment values of the aligned segments of the HDMs were similar across the different trematode peptides and predict that their C‐termini are similarly amphipathic perpendicular to their helical axes (Figure S4). To examine whether this unusual conservation of the helical hydrophobic region was reflected in the biological activity, synthetic peptides of 40 amino acids corresponding to the HDM.C2 peptide of the same trematode HDMs were examined for their anti‐inflammatory activity against LPS‐stimulated murine BMDMs and human THP‐1 macrophages (Figure 7D,E). While the peptides displayed anti‐inflammatory properties, there was variability in the potency of their immune regulatory activities, with FhHDM‐1.C2 showing the greatest inhibition of TNF production.

## 4 DISCUSSION

The objective of this study was to examine the structure–function relationship of the prototypical FhHDM‐1 peptide and employ this knowledge to identify the minimal amino acid sequence responsible for biotherapeutic activity, to enable the future design and synthesis of more efficacious peptide derivatives. To achieve this, we employed a series of in vitro assays to reflect the known mechanisms of FhHDM‐1 and to determine comparative potency of peptides. This approach identified a C‐terminal derivative, FhHDM‐1.C2, as the minimal bioactive peptide derivative in vitro. Validating this finding and verifying the predictive power of the chosen in vitro assays, the efficacy of the FhHDM‐1.C2 peptide translated to an in vivo model of immune‐mediated disease, namely, the EAE murine model. Similar to the full‐length FhHDM‐1, the FhHDM‐1.C2 derivative inhibited the onset of EAE when given prophylactically. More importantly, and relevant to disease treatment, we now show that treatment with the FhHDM‐1.C2 peptide prevented the progression of EAE when given therapeutically after disease onset.

FhHDM‐1 consists of two α‐helices separated by a short coil. The C‐terminal α‐helix contains a stretch of ~18 amino acids (ITEVITILLNRLTDRLEK) that forms a strongly amphipathic motif, which, by amino acid substitution designed to disrupt this amphipathicity, we have proven is required for the peptide to regulate the pro‐inflammatory responses of macrophages. Having previously characterized this as a region (FhHDM‐1.C1) which exhibited bioactive properties, cellular assays revealed that it alone was unable to fully replicate the immunomodulatory activity of the parent FhHDM‐1 peptide.^8^ However, in the present study, we discovered that an additional stretch of amino acids within the α‐helix (KARDR), before the short random coil, is required to mediate cellular binding and uptake of FhHDM‐1 by macrophages. Our bioinformatic analysis corroborates in silico predictions that this region exhibits the characteristics of a CPP. The presence of this motif provides an explanation for why we find the FhHDM‐1 peptide within the endosomal/lysosomal system, as uptake of most CPPs is mediated by endocytosis leading to their accumulation inside endosomes.^29^, ^30^ Furthermore, the coupling of the KARDR sequence to the amphipathic C‐terminal region of FhHDM‐1 peptide provides an explanation as to why the FhHDM‐1.C2 peptide exhibits greater efficacy as compared with FhHDM‐1.C1 which lacks the complete CPP sequence and has a reduced capacity to interact with macrophages and penetrate the cell membrane.

In all in vitro assays, the FhHDM‐1.C2 derivative was significantly more potent than the full‐length FhHDM‐1. While there is no apparent association between the structure–function to explain this difference, it may reflect the lack of requirement for enzymatic processing in the lysosome for the FhHDM‐1.C2. As mentioned, we have previously reported that FhHDM‐1 is cleaved by cysteine proteases within the lysosome, to release the C‐terminal derivative that subsequently inhibits vATPase.^8^ It is plausible, that the full‐length FhHDM‐1 parent has a 3D conformation that hinders a complete interaction with vATPase. In contrast, the shorter C2 derivative may be fully engaged, changing the physicochemical properties that can increase cellular translocation,^28^ and negating any required proteolytic processing for the release of the active component for optimal intracellular activity.

Although a direct comparison of potency was not performed in vivo, the FhHDM‐1.C2 mediated the same beneficial outcomes as we have previously reported for FhHDM‐1 in the same model of RR‐EAE, using the same prophylactic treatment regime.^9^ However, comparing these independent studies would suggest that the FhHDM‐1.C2 derivative is more efficacious. While 20% FhHDM‐1‐treated mice (of *n* = 15) showed no signs of disease in our previous report,^9^ 80% of the FhHDM‐1.C2 mice (of *n* = 5) in this current study remained disease‐free. Furthermore, FhHDM‐1.C2 also prevented the progression of disease in RR‐EAE when delivered after the onset of paralysis. Nonetheless, a side‐by‐side comparison would be worthwhile in the future to fully test comparative potency of the FhHDM‐1 and its C2 derivative administered at a range of doses and via different treatment regimes.

Importantly, and in contrast to many existing treatments for autoimmune/immune‐mediated diseases, the in vivo activity of FhHDM‐1.C2 was not associated with concomitant systemic immune suppression. The adaptive immune response to antigens remained intact and T cells retained their capacity to proliferate, produce cytokines in response to antigen stimulation, and to transfer disease from EAE conditioned mice to naïve recipients. The histological analysis of CNS tissue gives credence to speculate why these autoreactive immune cells, although present, do not mediate the progression of disease in the FhHDM‐1.C2 mice. While small accumulations of inflammatory cells were present in the brain tissue they were restricted to within the perivascular space; this observation could be explained by the regulation of macrophage activation by FhHDM‐1.C2, as pro‐inflammatory cytokines produced by infiltrating peripheral macrophages are known to activate the matrix metalloproteinases at the parenchymal border to promote leukocyte migration out of the perivascular cuff and into the brain tissue.^31^ In both EAE and MS, symptoms become apparent following leukocyte penetration of the basement membrane, indicating that penetration of the parenchymal border is a disease‐limiting step.^32^, ^33^ Furthermore, infiltrating monocytes are suggested to play a prominent role in driving disease pathogenesis. While the depletion of peripheral macrophages, or the specific inhibition of macrophage‐produced TNF, as occurs following FhHDM‐1.C2 treatment, does not impact the development of auto‐antigen specific T cells, the infiltration of these cytotoxic cells into the CNS parenchyma is inhibited, and thus demyelination and subsequent disease development is prevented.^34^, ^35^ Furthermore, monocyte depletion prior to the presentation of symptoms, delays the onset of EAE and results in less severe clinical scoring,^36^, ^37^ while depletion post‐onset also results in the prevention of disease progression.^36^, ^38^, ^39^ Therefore, the lack of penetration of inflammatory cells into the CNS tissues following treatment with FhHDM‐1.C2 supports a mechanism by which the peptide induces systemic macrophages/monocytes to regulate their pro‐inflammatory immune response, consequently preventing migration of auto‐destructive immune cells across the basement membrane of the blood–brain barrier. However, additional analysis of macrophage activity and function in situ, combined with the analysis of astrocyte and metalloprotease activity, and quantification of blood–brain barrier permeability, will be necessary to fully characterize this relationship. In addition, the adoptive transfer of monocytes/systemic macrophages from FhHDM‐1.C2‐treated mice will be required to definitively establish that it is these cells that are specifically mediating the potent, protective effect of the HDM peptides.

While the data presented here shows that FhHDM‐1.C2 clearly prevented the progression of disease, it remains to be determined whether, in the absence of infiltration leukocytes, repair mechanisms are also initiated by resident cells of the CNS to regenerate myelin and reverse clinical disease. Alternative animal models of disease (Cuprizone model) or in vitro systems (brain slice model) will be required to quantify a regenerative/repair effect of the HDM peptides.

Importantly, the ability to fine‐tune the inflammatory response of macrophages to mediate a protective effect is consistent with the decisive role for macrophages in the initiation and progression of other autoimmune diseases.^40^, ^41^ For example, in the nonobese diabetic mice (model of T1D), in which FhHDM‐1 also prevents development of disease, autoreactive T cells showed no auto‐reactivity against beta islet cells unless they are in a macrophage replete environment. The activation of T‐cell pathogenicity in this disease model, like EAE, is also dependent on the production of pro‐inflammatory cytokines by macrophages.^42^ However, the pathogenic role for pro‐inflammatory macrophages is not restricted to autoimmune conditions as these cells can also dictate the occurrence, development and persistence of conditions associated with chronic inflammation, such as IBD and nonalcoholic steatohepatitis,^43^, ^44^, ^45^, ^46^ suggesting a broad applicability for the FhHDM‐1 and FhHDM‐1.C2 peptides.

The results from this study also support an exploration into the efficacy of other members of the HDM family. Although, peptide derivatives that were designed according to the corresponding FhHDM‐1.C2 sequence in the other trematode HDMs displayed wide variation in their ability to modulate the activity of murine macrophages, we speculate that they possess equivalent therapeutic potential to the Fasciola HDM‐1.C2 peptide. As the full‐length versions were comparatively effective in their ability to regulate macrophage responses, it is most likely that differences in the potency of the C‐terminal peptides reflect variations in the minimal amino acid sequence comprising the functional “C2” derivative for each specific trematode HDM, in part due to variations in the sequence or length of their corresponding CPP motif. It is also possible that differences in the amino acid sequence of the HDM family of peptides alters the function of the peptides, which may correspond to a precise biological activity for each peptide in each parasite; with specific sequences complementing the tissue location in which the parasites resides and/or the type of resident immune cell/macrophages in these respective tissue compartments. Another property of these various trematode peptides that requires exploration is their capacity to enter cells, and if so, to which compartment they localize, since small changes in peptide charge or hydrophobicity, particularly in CPPs, can result in cytosolic or nucleolar localization, rather than endosome accumulation.^19^, ^47^, ^48^

The parasite HDM peptides have been evolutionary honed and optimized to interact with their host's immune cells, thereby ensuring longevity for the parasite while minimizing tissue damage for the host. Understanding this relationship between the structure of the HDM peptides derived from the multitude of trematode parasites and their tissue‐specific biological functions presents a unique opportunity to discover and re‐engineer a wide array of lead biotherapeutics for the treatment of human immune‐mediated diseases.

## AUTHOR CONTRIBUTIONS

R. Lalor, J.P. Dalton, S. Donnelly, and J. Greer conceived and designed the research; R. Lalor, A. Tanaka, J. Sheils, A. Dixit, S. Hoadley, E. Dufourd, J. To, S. Hamon, and S. Donnelly performed the research and acquired the data, R. Lalor, A. Tanaka, C.C. Taggart, S. Weldon, B. O'Brien, J. Greer, J. P. Dalton, and S. Donnelly analyzed and interpreted the data. All authors were involved in drafting and revising the manuscript.

## DISCLOSURES

The authors declare no conflicts of interest.

## Supporting information


Table S1.

Table S2.

Figure S1.

Figure S2.

Figure S3.

Figure S4.

Figure S5.

## Data Availability

The data that support the findings of this study are available in the Materials and Methods, Results, and/or Supplemental Material of this article.
