# Supplementary material for: An immunoregulatory amphipathic peptide derived from Fasciola hepatica helminth defense molecule (FhHDM‐1.C2) exhibits potent biotherapeutic activity in a murine model of multiple sclerosis
Source: FASEB J. 2025 Feb 14;39(4):e70380. doi: 10.1096/fj.202400793RR (PMC11826375; doi:10.1096/fj.202400793RR)
Supplement: Supplementary file 1 — Table S1. [file FSB2-39-e70380-s004.docx]

| **Class** | **Probability** | **Sequence** |
| --- | --- | --- |
| Non-CPP | 0.171848 | AMAYLAK |
| CPP | 0.746522 | RAMAYLAK |
| CPP | 0.762696 | DRAMAYLAK |
| CPP | 0.921527 | RDRAMAYLAK |
| CPP | 0.697418 | ARDRAMAYLAK |
| CPP | 0.973594 | KARDRAMAYLAK |
| CPP | 0.887798 | EKARDRAMAYLAK |
| Non-CPP | 0.398883 | YEKARDRAMAYLAK |
| Non-CPP | 0.111726 | AYEKARDRAMAYLAK |
| CPP | 0.848466 | KARDR |
| CPP | 0.861256 | KARDRA |
| Non-CPP | 0.428501 | KARDRAM |
| CPP | 0.911862 | KARDRAMA |
| CPP | 0.928411 | KARDRAMAY |
| CPP | 0.883935 | KARDRAMAYL |
| CPP | 0.778323 | KARDRAMAYLA |
| CPP | 0.973594 | KARDRAMAYLAK |
| CPP | 0.69358 | KARDRAMAYLAKD |
| Non-CPP | 0.043125 | KARDRAMAYLAKDNL |

**Supplementary Table 1.** CPP predictions of FhHDM-1 sequences
