# Supplementary material for: An immunoregulatory amphipathic peptide derived from Fasciola hepatica helminth defense molecule (FhHDM‐1.C2) exhibits potent biotherapeutic activity in a murine model of multiple sclerosis
Source: FASEB J. 2025 Feb 14;39(4):e70380. doi: 10.1096/fj.202400793RR (PMC11826375; doi:10.1096/fj.202400793RR)
Supplement: Supplementary file 2 — Table S2. [file FSB2-39-e70380-s001.docx]

**Supplementary Table 2.** Splenocytes from both PBS-treated mice and C2 peptide-treated mice passively transfer EAE.

| **Splenocytes from** | **Incidence of EAE** | **Peak score** | **Day of onset** |
| --- | --- | --- | --- |
| PBS-treated | 5/5 | 1.2 ± 0.1 | 21.8 ± 5.2 |
| FhHDM-1.C2 peptide-treated | 5/5 | 1.5 ± 0.3 | 13.0 ± 1.8 |
