# Supplementary material for: An immunoregulatory amphipathic peptide derived from Fasciola hepatica helminth defense molecule (FhHDM‐1.C2) exhibits potent biotherapeutic activity in a murine model of multiple sclerosis
Source: FASEB J. 2025 Feb 14;39(4):e70380. doi: 10.1096/fj.202400793RR (PMC11826375; doi:10.1096/fj.202400793RR)
Supplement: Supplementary file 3 — Figure S1. [file FSB2-39-e70380-s003.pdf]

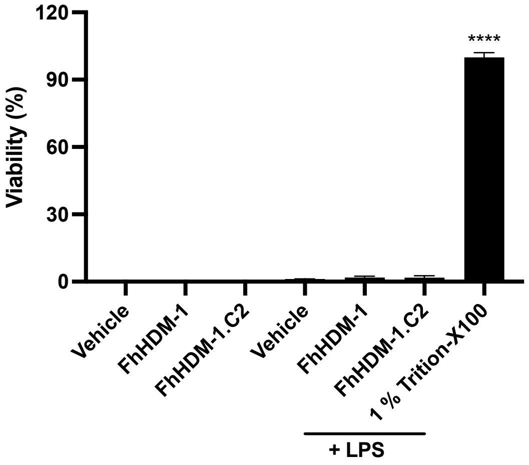

Supplementary Figure 1. Treatment of macrophages with bacterial LPS and HDM peptides does not reduce cell viability. Effects of stimulants on cell viability were determined by the quantification of LDH release from BMDMs treated with vehicle, FhHDM-1 or FhHDM-1.C2 (10 μM) with and without LPS (100 ηM) for 18 hrs. Data presented as the mean ± S.D. and analysed using ANOVA with Tukey's multiple comparison test . Stars indicate degree of significance when compared to vehicle controls. ****p <0.001.
