# Supplementary material for: An immunoregulatory amphipathic peptide derived from Fasciola hepatica helminth defense molecule (FhHDM‐1.C2) exhibits potent biotherapeutic activity in a murine model of multiple sclerosis
Source: FASEB J. 2025 Feb 14;39(4):e70380. doi: 10.1096/fj.202400793RR (PMC11826375; doi:10.1096/fj.202400793RR)
Supplement: Supplementary file 4 — Figure S2. [file FSB2-39-e70380-s005.pdf]

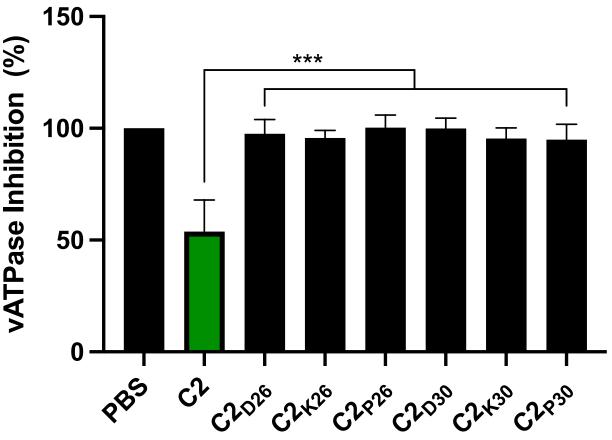

Supplementary Figure 2. Disrupting the the amphipathic region of the FhHDM-1.C2 peptide removes the capacity to inhibit vATPase. FhHDM-1.C2 or FhHDM-1.C2 analogues (13 μM) with substitutions of leucine residues at position 26 or 30 with aspartic acid (D), lysine (K) or proline (P) were pre-incubated with lysosomal enriched fractions prior to incubation with the ATP substrate. The figure shows the inhibitory effects of the HDMs as a percentage reduction in ATPase activity compared to vehicle treated control. Data are presented as the mean ± S.D. of 3 independent experiments and were analysed using a repeated measures ANOVA with Tukey's multiple comparison test. Stars indicate degree of significance when FhHDM-1.C2 analogues compared to FhHDM-1.C2. ***$p < 0.001$.
