# Supplementary material for: An immunoregulatory amphipathic peptide derived from Fasciola hepatica helminth defense molecule (FhHDM‐1.C2) exhibits potent biotherapeutic activity in a murine model of multiple sclerosis
Source: FASEB J. 2025 Feb 14;39(4):e70380. doi: 10.1096/fj.202400793RR (PMC11826375; doi:10.1096/fj.202400793RR)
Supplement: Supplementary file 5 — Figure S3. [file FSB2-39-e70380-s007.pdf]

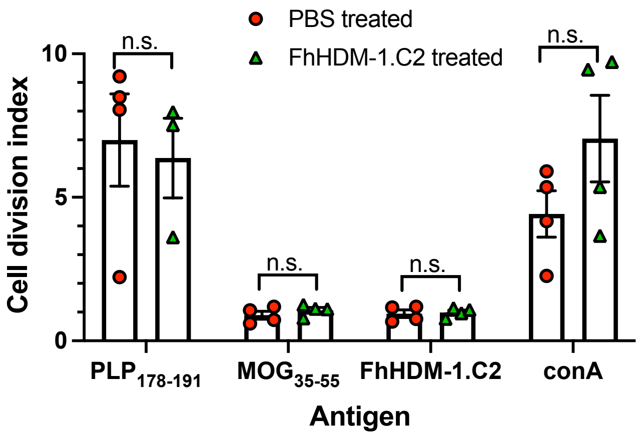

Supplementary Figure 3. FhHDM-1.C2 does not alter T cell proliferation. $PLP_{178\text{-}191}$ immunized mice were treated with PBS or FhHDM-1.C2 as per the prophylactic treatment protocol. Three days after the final treatment, lymph nodes were removed from the mice, lymph node cells were labelled with cell-trace violet (CTV) and stimulated *in vitro* with no antigen, $PLP_{178\text{-}191}$, $MOG_{35\text{-}55}$, FhHDM-1.C2 or ConA for 3 days. Cells were then harvested, labelled with PE-labelled anti-CD4 antibody and assessed for proliferation of $CD4^{+}$ T cells (indicated by decreased CTV staining) by flow cytometry. The cell division index was determined from the # of the proliferating cells in response to antigen / # of proliferating cells in the no antigen control group. Bars represent mean and S.D.
