# Supplementary material for: An immunoregulatory amphipathic peptide derived from Fasciola hepatica helminth defense molecule (FhHDM‐1.C2) exhibits potent biotherapeutic activity in a murine model of multiple sclerosis
Source: FASEB J. 2025 Feb 14;39(4):e70380. doi: 10.1096/fj.202400793RR (PMC11826375; doi:10.1096/fj.202400793RR)
Supplement: Supplementary file 6 — Figure S4. [file FSB2-39-e70380-s006.pdf]

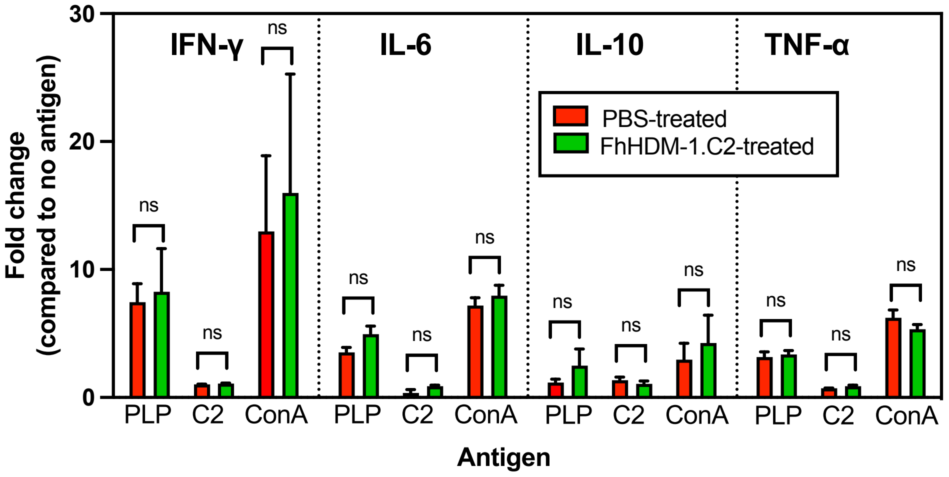

Supplementary Figure 4. FhHDM-1.C2 does not inhibit encephalitogenic T cell reactivity. $PLP_{178\text{-}191}$ immunized mice were treated with PBS or FhHDM-1.C2 as per the therapeutic treatment protocol. Fifty days after the final treatment, lymph nodes were removed from the mice and lymph node cells were stimulated *in vitro* with no antigen, $PLP_{178\text{-}191}$, FhHDM-1.C2 or ConA for 6 days. Culture supernatants were removed and immediately tested (in duplicate) for cytokine levels using mouse cytokine kits for IFN-γ, IL-6, IL-10 or TNF from elisakit.com, as per the manufacturer's protocols. The figure shows fold changes (compared to no antigen controls) in levels of cytokines produced by the LNC from mice treated with PBS (red bars) or FhHDM-1.C2 (green bars), in response to stimulation to the immunizing antigen $PLP_{178\text{-}191}$ (PLP), FhHDM-1.C2 (C2) or ConA. Each bar represents the results of LNC cultures from 4 different mice.
