# Supplementary material for: An immunoregulatory amphipathic peptide derived from Fasciola hepatica helminth defense molecule (FhHDM‐1.C2) exhibits potent biotherapeutic activity in a murine model of multiple sclerosis
Source: FASEB J. 2025 Feb 14;39(4):e70380. doi: 10.1096/fj.202400793RR (PMC11826375; doi:10.1096/fj.202400793RR)
Supplement: Supplementary file 7 — Figure S5. [file FSB2-39-e70380-s002.pdf]

FhHDM-1	Sequence	SEESREKLRESGRKMVKALRDAVTKAYEKARDRAMAYLAKDNLGEKITEVITILLNRLTDRLEKYAGN
	Prediction	CCHHHHHHHHHHHHHHHHHHHHHHHHHHHHHHHHHHHHHHCCHHHHHHHHHHHHHHHHHHHHHHHCCC
	Conf.Score	906689999988899999999999999999999999976207789999999999999999999877369
		H:Helix; S:Strand; C:Coil
CsHDM-1	Sequence	SEETRAKLRESGQKLWTAVVAAARKCAERVRQRIEEYLEKDNLGEKIAEVVKILSERLTKRIETYVGE
	Prediction	CCHHHHHHHHHHHHHHHHHHHHHHHHHHHHHHHHHHHHHHCCHHHHHHHHHHHHHHHHHHHHHHHHCC
	Conf.Score	9167899999879999999999999999999999997743537999999999999998999867258
		H:Helix; S:Strand; C:Coil
PwHDM-1	Sequence	DSQEQLRETGKNLYEAIRKAVMKIAQKCKAKIDAYLEKDGLGDKISEVIQILLKRLTDRIEKYVEN
	Prediction	CHHHHHHHHHHHHHHHHHHHHHHHHHHHHHHHHHHHHHCCHHHHHHHHHHHHHHHHHHHHHHHCCC
	Conf.Score	928799997799999999999999999999999997666347899999999999998999857378
		H:Helix; S:Strand; C:Coil
OvHDM-1	Sequence	NEETRAKLRESGQKLWGAIMSAAKKCADRVKQRIEEYLEKDGLGEKLADVIKILAERLTKRMETYVKE
	Prediction	CHHHHHHHHHHHHHHHHHHHHHHHHHHHHHHHHHHHHHHHCCHHHHHHHHHHHHHHHHHHHHHHHCCC
	Conf.Score	978799999889999999999999999999999997555547899999999999998999866178
		H:Helix; S:Strand; C:Coil
SjHDM-1	Sequence	ENLRFQLQKTLMDTGEKFKTLSLRLLTRCRNRVREYFKQDDLGEKIAEVLLIFLQRLNRRLEKYLSRPE
	Prediction	CCHHHHHHHHHHHHHHHHHHHHHHHHHHHHHHHHHHHHHCCHHHHHHHHHHHHHHHHHHHHHHHCCCCC
	Conf.Score	948899999999989999999999999999999998754374799999999999999999997548998
		H:Helix; S:Strand; C:Coil
SjHDM-2	Sequence	ENSRLLLQKALMDTGEKFKTLSLRLLARCRDRVREYFKQDGLGEKLAEVLLILLQRLNRRLEKYLPRSE
	Prediction	CCHHHHHHHHHHHHHHHHHHHHHHHHHHHHHHHHHHHHHCCHHHHHHHHHHHHHHHHHHHHHHHCCCCC
	Conf.Score	948899999999989999999999999999999998754374799999999999999999986168999
		H:Helix; S:Strand; C:Coil
SmHDM	Sequence	VTAESQASQKELFTESVKLWKSITELWKRFEHNCRVKIRKYLEEDNLGEKLAAVVSIYVKRLNKRLDMRLSEDRAE
	Prediction	CCCCHHHHHHHHHHHHHHHHHHHHHHHHHHHHHHHHHHHHHHHHCCHHHHHHHHHHHHHHHHHHHHHHHHHHHHCC
	Conf.Score	9775077899999999999999999999999999999999987620758999999999999999999887575049
		H:Helix; S:Strand; C:Coil

Supplementary Figure 5. *In Silico* Structural analyses of Helminth Defence Molecules (HDMs). Primary sequences of HDMs were uploaded to I-TASSER to produce secondary structural predictions. (Yang & Zhang 2015; https://zhanglab.ccmb.med.umich.edu/I-TASSER/)
